# Personal

**Published:** 1901-08

**Authors:** 


### PERSONAL.

Dr. Adam H. Wright, of Toronto, editor *Canadian Practitioner and Review*, recently spent three months in Europe. He gives an interesting account of affairs, medical and otherwise, in Great Britain and on the continent, in his magazine for July, 1901.

Dr. A. A. Hubbell, of Buffalo, for several years associate editor of this journal, has resigned from the editorial staff, on account of increasing professional demands upon his time. Dr. Hubbell is an efficient worker in the field of medical periodical literature from whom we part with regret.

Dr. Lorenzo Burrows, Jr., of Buffalo, has been elected by the staff of the Buffalo General Hospital, as ophthalmologist to that institution to fill the vacancy created by the death of Dr. Frank W. Abbott. Dr. Burrows also has been appointed ophthalmologist and otologist at the Fresh Air Mission Hospital, located at Athol Springs, on the Lake Shore.

---

Dr. Ernest L. Ruffner, of Buffalo, has been appointed, after due examination, an assistant surgeon (first lieutenant) in the U. S. Army, and has been assigned to temporary duty at Columbus, O. Dr. Ruffner held a similar position in the 65th Regiment N.Y. Vols., during the war with Spain, with the rank of captain. On advertising page xxiv., will be found an offer by Dr. Ruffner to sell an electrical battery.

---

Dr. Julius Ullman, of Buffalo, sometime ago was appointed instructor in clinical medicine and assistant in the bacteriological laboratory at the University of Buffalo. Through oversight in preparing copy for the printer, Dr. Ullman's name was omitted from the college announcement for 1901. Dr. Ullman has not been dropped from the list, but he still continues to perform the functions above named.

---

Dr. R. T. Rolph, of Castle Creek Sanitarium, Hot Springs, Arizona, formerly of Dunkirk, N.Y., is spending a few weeks at his old home. Incidentally, he is occasionally paying a visit to the Pan-American exposition at Buffalo, and the Journal is pleased to welcome him among its many Pan-American visitors.

---

Dr. B. T. Whitmore, of New York, who has for so many years been associated with Messrs. Parke, Davis & Co., in their scientific department, recently went to Europe to attend the British Tuberculosis Congress. In the early part of the season Dr. Whitmore and his family visited the Pan-American exposition at Buffalo, where a number of delightful days were passed.

---

Dr. J. N. Hurty, of Indianapolis, secretary of the State Board of Health of Indiana, recently spent a week or two at Buffalo for rest and recuperation, and incidentally to see the Pan-American exposition. He was accompanied by his wife and son.

Dr. Thomas E. Spalding, U. of B., '01, has located for the practice of his profession at Salamanca, N.Y.

---

Dr. L. G. Hanley, of Buffalo, president of the medical staff of the Hospital of the Sisters of Charity, lately went to Emmettsburg, Md., to attend the opening of the Normal Hospital of the

Sisters of Charity in that city. Prominent physicians from New York, Chicago, St. Louis and other cities were present. Dr. Hanley during his visit of a week delivered a course of lectures at the hospital.

---

Dr. Willis G. Gregory, of Buffalo, has established a truss department at his pharmacy in The Genesee, 530 Main Street, where special attention will be given the important work of truss-fitting in all its various phases.

---

Dr. Margaret A. Cleaves, of New York, has recently been appointed the American editor of the *Journal of Physical Therapeutics*, a Quarterly International Review published at London.

---

Dr. J. D. Griffith, of Kansas City, president of the Missouri State Medical Association, visited Buffalo and the exposition recently. Dr. Griffith is one of the prominent surgeons of the west and has promised to visit Buffalo again before the exposition closes.

---

Governor-General Wood, of Cuba, who has been afflicted with typhoid fever, is reported convalescent. He will visit the United States and spend a few weeks in New England for recuperation.

